# Study protocol: evaluation of specialized outpatient palliative care (SOPC) in the German state of Hesse (ELSAH study) – work package II: palliative care for pediatric patients

**DOI:** 10.1186/s12904-017-0268-y

**Published:** 2018-01-05

**Authors:** Lisa-R. Ulrich, Dania Gruber, Michaela Hach, Stefan Boesner, Joerg Haasenritter, Katrin Kuss, Hannah Seipp, Ferdinand M. Gerlach, Antje Erler

**Affiliations:** 1 Institute of General Practice, Goethe-University Frankfurt, Frankfurt, Germany; 2 Professional Association of Specialized Outpatient Palliative Care in Hesse, Wiesbaden, Germany; 3 Department of General Practice and Family Medicine, Philipps-University of Marburg, Marburg, Germany

**Keywords:** Palliative care [MeSH], Pediatrics [MeSH], Qualitative research [MeSH], Needs assessment [MeSH], Mixed methods

## Abstract

**Background:**

In 2007, the European Association of Palliative Care (EAPC) provided a comprehensive set of recommendations and standards for the provision of adequate pediatric palliative care. A number of studies have shown deficits in pediatric palliative care compared to EAPC standards. In Germany, pediatric palliative care patients can be referred to specialized outpatient palliative care (SOPC) services, which are known to enhance quality of life, e.g. by avoiding hospitalization. However, current regulations for the provision of SOPC in Germany do not account for the different circumstances and needs of children and their families compared to adult palliative care patients. The “Evaluation of specialized outpatient palliative care (SOPC) in the German state of Hesse (ELSAH)” study aims to perform a needs assessment for pediatric patients (children, adolescents and young adults) receiving SOPC. This paper presents the study protocol for this assessment (work package II).

**Methods/Design:**

The study uses a sequential mixed-methods study design with a focus on qualitative research. Data collection from professional and family caregivers and, as far as possible, pediatric patients, will involve both a written questionnaire based on European recommendations for pediatric palliative care, and semi-structured interviews. Additionally, professional caregivers will take part in focus group discussions and participatory observations. Interviews and focus groups will be tape- or video-recorded, transcribed verbatim and analyzed in accordance with the principles of grounded theory (interviews) and content analysis (focus groups). A structured field note template will be used to record notes taken during the participatory observations. Statistical Package for Social Sciences (SPSS, version 22 or higher) will be used for descriptive statistical analyses. The qualitative data analyses will be software-assisted by MAXQDA (version 12 or higher).

**Discussion:**

This study will provide important information on what matters most to family caregivers and pediatric patients receiving SOPC. The results will add valuable knowledge to the criteria that distinguish SOPC for pediatric from SOPC for adult patients, and will provide an indication of how the German SOPC rule of procedure can be optimized to satisfy the special needs of pediatric patients.

**Trial registration:**

Internet Portal of the German Clinical Trials Register (www.germanctr.de, DRKS-ID: DRKS00012431).

## Background

In Germany, approximately 23,000 children and adolescents [1] and 87,460 adults [2] are suffering from life-limiting conditions. Both adult and pediatric patients receiving palliative care can be referred to the specialized outpatient palliative care (SOPC) program. SOPC is provided by an interprofessional team (physicians, palliative care nurses, psychologists, social workers) in the patient’s family environment and includes palliative medical and nursing care, a 24/7 on-call service, psychosocial support and coordination of care in cooperation with local health care providers (family physicians/pediatricians) [2]. SOPC was introduced in 2007 to alleviate suffering and enhance quality of life in patients and their relatives, e.g. by avoiding hospitalization [2]. Requirements and reimbursement for SOPC are subject to a specific rule of procedure determined by the Federal Joint Committee (G-BA), the highest decision-making body of the joint self-government of physicians, dentists, hospitals and health insurance funds in Germany [3]. The rule of procedure regulating the delivery of SOPC apply to both adult and pediatric patients in palliative care. However, there are a number of significant differences between the two groups of patients. While the majority of adult palliative care patients suffer from oncologic diseases, most pediatric patients cared for by SOPC teams have congenital diseases [4]. Most of these diseases are complex, rare and often associated with cognitive disabilities. In addition, the wide range of ages and developmental stages of the patients treated under the umbrella of SOPC for pediatric patients are a challenge [5]. SOPC for adults typically commences towards the end of the disease course and close to death [4]. The course of disease in children is often characterized by recurrent crises interspersed by recoveries that render further SOPC unnecessary until the next crisis occurs. Thus, pediatric SOPC requires a more flexible approach, e.g. by adapting intensity of care depending on the changing needs of the patient [4]. In palliative care in general, the involvement of the family plays an important role. While the whole family is affected when an adult family member is diagnosed with a life-limiting condition, their involvement and suffering will be greater when a child’s life is at stake [6]. As the responsible caregivers, parents are the main contact persons for pediatric SOPC teams [7], especially for young children and children unable to communicate verbally. A 2010 study showed that legal and financial regulations in Germany do not sufficiently take into account the needs of pediatric patients receiving palliative care [8]. This problem is not confined to Germany. Although in 2007 the European Association of Palliative Care (EAPC) provided a comprehensive set of recommendations and standards for the provision of adequate palliative care to pediatric patients [9, 10], international studies also highlighted deficits. These particularly concern the delivery of family-centered care [11–13] that adequately addresses siblings’ needs [14], and provides psychosocial support and follow-up care for families [15, 16].

In this context, our study “Evaluation of specialized outpatient palliative care in the German state of Hesse (ELSAH)” aims to clearly define the special needs and requirements that distinguish SOPC for children and adolescents from SOPC for adults. It focuses on a needs assessment for pediatric patients (children, adolescents and young adults) receiving SOPC. The results of the study will be used to adapt the current German rule of procedure for SOPC to suit the special needs of palliative care for pediatric patients. Research questions addressed in this study are:

- Taking the current situation in the German state of Hesse as an example, to what extent is palliative care for pediatric patients provided in accordance with European (EAPC) recommendations and standards? [9] What works well and where is there room for improvement?
- What special needs do pediatric patients (children, adolescents and young adults) have, and how does SOPC for children differ from SOPC for adults?

Besides these research questions, another ELSAH work package (WP I) will focus on defining the term ‘quality of care’ within the context of SOPC, and defining and evaluating an instrument for measuring quality of care in adult patients receiving SOPC. The Department of General Practice and Family Medicine of Philipps-University of Marburg, Germany, will be responsible for WP I, and the study protocol will be published separately. This paper presents the study protocol for a needs assessment for pediatric patients receiving SOPC (WP II).

## Methods/Design

### Design and setting

**Fig. 1:**
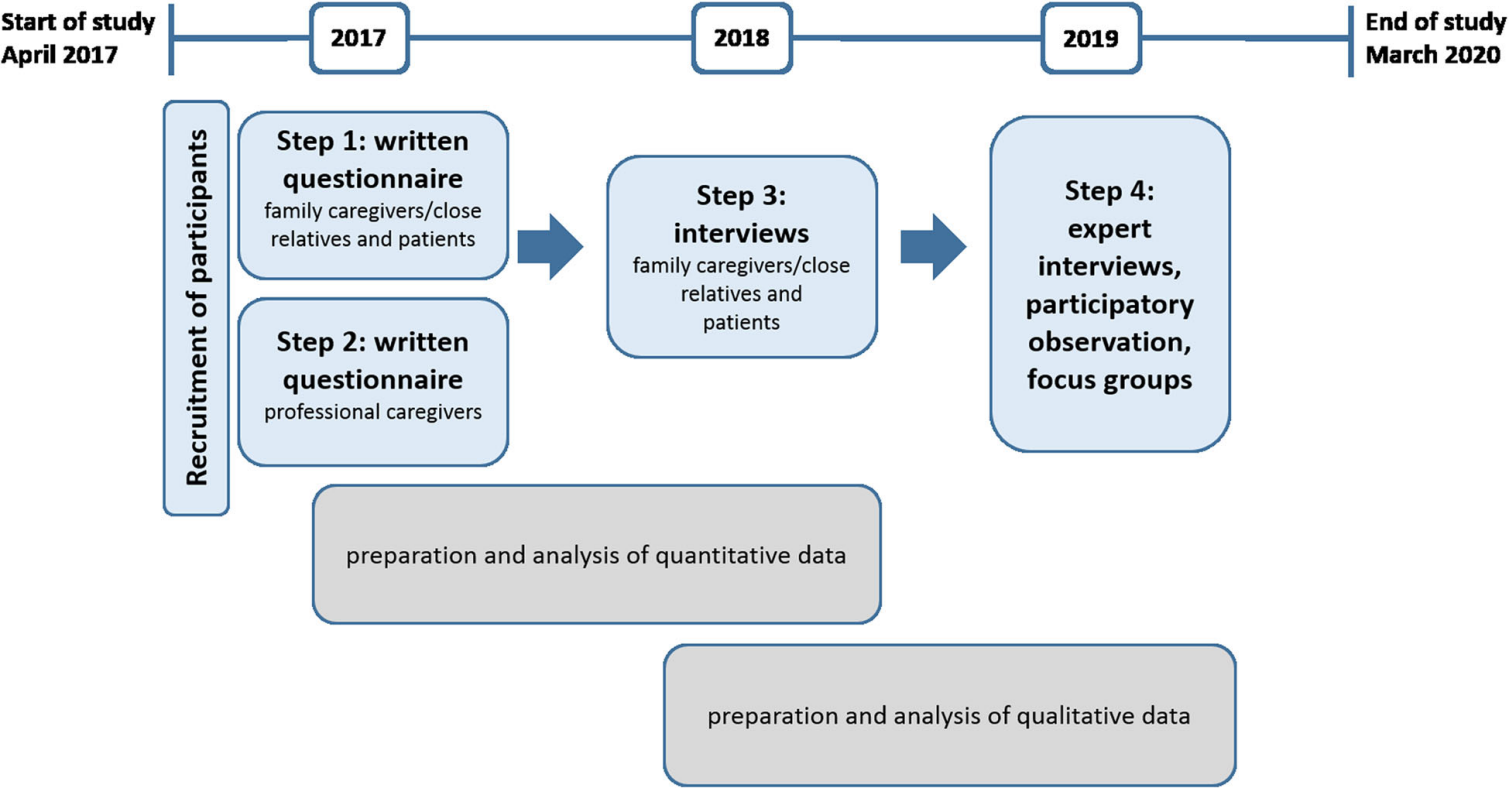
Timeline for the ELSAH study, WP II

This study will be conducted in the federal state of Hesse, Germany. It will use a sequential mixed-methods design [17], whereby we will focus on qualitative methods [18]. The quantitative approach will consist of a questionnaire survey of pediatric SOPC patients and their families, as well as members of the SOPC team and other professional caregivers (step 1 and 2). The qualitative approach will entail narrative interviews with pediatric patients receiving SOPC and their families (step 3), as well as semi-structured expert interviews, focus group discussions and participatory observation involving the SOPC teams and other professional caregivers (step 4). The use of different approaches will permit the triangulation of study results [19]. The results of the quantitative analyses will used to specify the qualitative data collection. Including recruitment, data collection, and analysis, the entire study will last 36 months (April 2017 to March 2020). A timeline for the ELSAH study can be found in Fig. 1.

### Sample and procedure

Currently, there are three pediatric SOPC teams in Hesse [20]. These teams are members of the Professional Association of SOPC in Hesse, which is a cooperation partner in our study. As the SOPC teams have a close relationship with pediatric patients, their families and professional caregivers, the Association will ask them to recruit study participants. Prior to recruitment, the SOPC teams will attend a one-day training workshop held by the study team of the Institute of General Practice at Goethe-University Frankfurt. Eligible study participants are pediatric palliative care patients in Hesse (*N* = 129 in 2015), their family members, and professional caregivers, i.e. pediatric SOPC teams (*N* = 3 in 2017), children’s hospices (*N* = 1 in 2017) and their special outpatient services (*N* = 9 in 2017), and socio-pediatric centers (*N* = 7 in 2017). Families in which SOPC has been suspended or the child recently died are also eligible for study participation.

Inclusion criteria for pediatric palliative care patients are:

- Age 7 to ≤27 years^1^ (able to give informed consent)
- Signed informed consent form; in the case of minors to include an additional informed consent form signed by the child’s custodians (usually both parents)

Inclusion criteria for family members are:

- Age ≥ 18 years (able to give informed consent)
- Age of siblings ≥7 years
- Signed informed consent form; in the case of minors to include an additional informed consent form signed by the child’s custodians (usually both parents)

Inclusion criteria for professional caregivers and participating SOPC teams are:

- Signed informed consent form
- Professional caregivers and their cooperation partners have official accreditation for provision of SOPC (in accordance with § 132d SGB V)
- SOPC was not provided before 01/01/2015
- SOPC is/was performed in Hesse

Exclusion criteria for all study participants are:

- insufficient command of the German language

Eligible study participants have the option to sign a form permitting the study team to contact them, or they can contact the study team themselves. After contact has been established, the study team will send all essential study information to the participant (e.g. informed consent form, questionnaires, stamped addressed envelope to be returned to the study team).

### Sample size

The recruitment of participants in such a vulnerable and sensitive setting as pediatric palliative care can be challenging, and research may represent a substantial burden on this population [21]. The task of filling out a questionnaire and the interview situation itself may be stressful for patients and their family caregivers and time-consuming for the pediatric SOPC team. Furthermore, patients may die during the study as a result of their life-limiting conditions. We have therefore chosen to use convenience sampling [22].

#### Steps 1 and 2: Questionnaire survey of pediatric patients, their families and professional caregivers

We do not consider it a condition that the sample of surveyed participants meets the requirements of a statistical power calculation. In line with convenience/availability sampling [22], all eligible study participants will be asked to participate and everyone who provides informed consent will be included in the study.

#### Steps 3 and step 4: Narrative interviews with pediatric patients and their families, expert interviews and focus groups with professional caregivers, participatory observation

The number of narrative interviews and expert interviews will depend on the theoretical saturation of the collected data [23] and will follow purposeful sampling [24]. Rather than to conduct as many as possible, our aim is to conduct interviews that deal as far as possible with issues of central importance to our research [25]. We expect to carry out 20 to 30 interviews for each study group and aim to interview participants from as broad a selection of professions and regions of Hesse as possible. We will also conduct three focus groups, each involving approximately 10 participants. We plan at least one participatory observation with each of the three SOPC teams in Hesse.

### Data collection and tools

#### Step 1: Questionnaire survey for pediatric patients and their families

Quantitative data collection will involve a self-administered questionnaire developed on the basis of recommendations for pediatric palliative care defined by the European Association of Palliative Care (EAPC) [9]. We will ask pediatric patients, their siblings and parents for their views on the extent to which palliative care is provided in accordance with EAPC recommendations (e.g. provision of care, care coordination, bereavement, communication and decision-making) and where there is room for improvement in the provision of SOPC for children and adolescents. Participants will be asked to provide personal details such as age and gender. The data will be analyzed descriptively using the Statistical Package for Social Sciences (SPSS, version 22 or higher) and suitable statistical parameters such as frequencies, means, and standard deviation. The questionnaire will be pilot tested before finalization and available in different versions depending on the age and development stage of the participant.

#### Step 2: Questionnaire survey for professional caregivers (including SOPC teams)

In step 2, data collection and analysis will be the same as described in step 1. However, the questionnaire will be adapted to accommodate the different perspectives of this group.

#### Step 3: Narrative interviews with pediatric patients and their families

Based on the responses to the questionnaire, we will develop and pilot semi-structured, mostly narrative interviews. In the interviews, we will ask family caregivers and, in as far as it is possible and acceptable to parents, pediatric patients about their special needs, as well as what distinguishes SOPC for pediatric from SOPC for adult patients. We will use a short questionnaire to collect socio-demographic data prior to the interviews. The interviews will be tape-recorded, transcribed verbatim and analyzed in accordance with the principles of grounded theory [26]. The software program MAXQDA (version 12 or higher) will be used to support the analysis. All study team researchers involved in conducting interviews will participate in a half-day training course on how to conduct research when participants are vulnerable. Upon request, a professional psychologist will also be available to provide supervision for study team members during the whole study period.

#### Step 4: Expert interviews, focus group discussions, and participatory observation with professional caregivers

Based on the responses to the questionnaire, we will develop and pilot an interview guide for expert interviews with members of the three pediatric SOPC teams, the children’s hospices and their special outpatient services, and socio-pediatric centers. The interview guide will cover the following broad topics:

- The extent to which palliative treatment goals are reached, including deficits in the implementation of palliative care standards, and possible causes.
- The type and quality of communication, coordination and cooperation between care providers, patients and their families resulting from the use of multidisciplinary SOPC teams.
- The support and relief services provided as part of palliative care (e.g. respite care services, psychosocial support/support for siblings).
- The special needs of children and adolescents that are receiving SOPC.

We will use a short questionnaire to collect socio-demographic data prior to the interview. The expert interviews will generally be conducted by phone, and tape-recorded. The interviews will be transcribed verbatim and analyzed in accordance with the principles of grounded theory [26]. Furthermore, we will conduct focus group discussions with pediatric SOPC teams, caregivers and special outpatient services at children’s hospices, and socio-pediatric centers, and we will use elements of content analysis [18] to analyze them after video recording. The expert interviews and focus group discussions will be supplemented by participatory observation [27], i.e. members of the study team will accompany the pediatric SOPC teams and observe their work with pediatric patients directly [28]. Notes will be collected using a structured field note template [29]. Participants in expert interviews and focus group discussions will receive a financial incentive to cover expenses (e.g. travel costs).

### Data management

All participant data will be pseudonymized. Retrospective identification of study participants will only be possible by means of a master database. Primary data (e.g. tape recordings, questionnaires) and the master database will be password protected at the Institute of General Practice in Frankfurt. The master database will be deleted at the end of the study, while all other study documents will be deleted after 10 years. All collected data will be anonymized prior to publication. The project will be carried out in accordance with the Proposals for Safeguarding Good Scientific Practice prepared by the German Research Foundation (DFG) [30].

## Discussion

Although SOPC in Germany has been carried out in accordance with a specific rule of procedure since 2007, the special needs of pediatric palliative care patients remain to be defined. Several German studies have begun to evaluate SOPC for pediatric patients from the family caregiver’s perspective, for example in the federal states of North Rhine-Westphalia [8, 31], Lower Saxony [15] and Bavaria [7, 32]. All of these studies recommend further research into such topics as psychosocial support, grief work, follow-up care after a child’s death, and needs assessments for siblings. These topics are among those that will be evaluated in our study, and the results are expected to enable the German rule of procedure for SOPC to be improved. For this reason, we would also like the siblings of sick children and adolescents to participate in the study. As part of the ELSAH study, our foremost aim is to address research questions in the field of pediatric palliative care that have not yet been answered satisfactorily. These include the identification of the support and relief services that are provided as part of palliative care (e.g. respite care services, psychosocial support/ support for siblings) and the special needs of children and adolescents receiving SOPC [33]. We will include viewpoints from all persons involved in pediatric SOPC and try to combine them in a way that enables SOPC for pediatric patients to be optimized. The use of interviews and participant observation will also provide a multi-perspective view that considers individual viewpoints, while also taking into account interactions between participants. In doing so, the study will provide the most comprehensive picture of the research topic yet available. The use of a mixed-methods design would appear to be suitable for this purpose [34], and a focus on qualitative methods will permit needs to be assessed in greater depth.

The ELSAH study will take place in an extremely challenging setting [21]. Although we would like to include young children and adolescents in our research, it will be necessary to take into consideration that they are all suffering from life-limiting diseases. Assessing a child’s ability to understand and make decisions whether to participate in research is difficult [35]. In the ELSAH study, every study participant will have to sign an informed consent form, and in the case of minors, an additional informed consent form will have to be signed by the child’s custodians (usually both parents). A total of 7 appropriately tailored versions of the informed consent form are available which take into account the different ages and development stages of participants, as well as individual family backgrounds (patient, family member, or professional caregiver). Ultimately, it will be the parents’ decision whether minors should participate in our study. We are fully aware that this may limit the size of the sample of family caregivers and pediatric patients. An unwillingness to confide may also be a problem when researchers are interviewing family members, children and adolescents at the same time [36]. In the ELSAH study, the decision whether to conduct interviews with family caregivers and patients separately or together will be made by the child. The interviewers will treat interviews conducted with individual patients confidentially and will not discuss them with his/her parents and/or his/her professional caregivers without the patient’s permission. We aim to conduct the needs assessments with the support of the pediatric patients themselves, but will also have to deal with cases in which self-assessment is impossible (e.g. because the child is not able to communicate verbally due to his disease). Our study population will therefore also consist of close caregivers, such as parents, siblings and SOPC team members. The ELSAH study will take place in the federal state of Hesse so inferences for the whole of Germany can only be drawn to a limited extent. We will recruit by means of convenience sampling [22], so our data may be biased by self-selection. Furthermore, as insufficient command of the German language is a criterion for exclusion, the participation rate of families from non-German speaking countries, possibly with different beliefs, value systems and religions, may be rather low. However, as cultural and religious backgrounds often influence attitudes towards illness and death, particularly in the case of end-of-life care for children [37], we will recruit persons from different cultural and religious backgrounds wherever possible.

This study will provide important information on what matters most for family caregivers and pediatric patients receiving SOPC. The results will add valuable knowledge about what distinguishes SOPC for pediatric from SOPC for adult patients. We aim to find out how the German specific rule of procedure for SOPC can be optimized to take into account the special needs of pediatric patients and, on this basis, to make recommendations how pediatric SOPC can be improved, mainly, but not exclusively, in Germany.

## Abbreviations

DFG: German Research Foundation (Deutsche Forschungsgemeinschaft)
DRKS: German Clinical Trials Register (Deutsches Register Klinischer Studien)
EAPC: European Association of Palliative Care
G-BA: Federal Joint Committee (Gemeinsamer Bundesausschuss)
SGB: Book of the Social Code (Sozialgesetzbuch)
SOPC: Specialized outpatient palliative care
WP: Work package

## Footnotes

1 The indicated age groups refer to recommendations provided by German law (§7(1) SGB VIII).
